# Exclusion and Repulsion of *Popillia japonica* (Coleoptera: Scarabaeidae) Using Selected Coverings on High Tunnel Structures for Primocane Red Raspberry

**DOI:** 10.3390/insects13090771

**Published:** 2022-08-26

**Authors:** Eric C. Burkness, Dominique N. Ebbenga, Adam G. Toninato, William D. Hutchison

**Affiliations:** Department of Entomology, University of Minnesota, 1980 Folwell Avenue, Saint Paul, MN 55108, USA

**Keywords:** pest exclusion, high tunnels, integrated pest management (IPM), raspberry

## Abstract

**Simple Summary:**

The Japanese beetle, *Popillia japonica* Newman, has become an invasive pest of increasing concern for several high-value berry crops in the Midwest region of the U.S. since 2010. Due to the feeding injury caused by adult beetles, many producers have increased their use of foliar insecticides to manage *P. japonica*. With a goal to develop sustainable Integrated Pest Management (IPM) systems for raspberry production, we initiated a 3-year study to examine the potential for various high-tunnel production systems to suppress *P. japonica* populations, and thus minimize insecticide use in primocane (autumn maturing) raspberry. During each year of the study, we observed significant reductions in *P. japonica* beetle infestations (*p* < 0.05), under the high-tunnel, covered systems, compared to nearby open plots of raspberry.

**Abstract:**

In temperate climates, there has been an increasing interest by fruit growers to implement the use of high tunnels, using a variety of coverings, to extend the season for fruit production. High tunnels also provide an opportunity to enhance insect pest management, via physical exclusion, and thus support reductions in insecticide use. Due to increasing pest pressure by the Japanese beetle, *Popillia japonica* Newman, in Midwest U.S. raspberry, a 3-year study (2017–2019) was conducted to evaluate the efficacy of selected high tunnel coverings to suppress adult beetle populations and minimize adult feeding injury. During each year of the study, *P. japonica* adult beetles were significantly reduced under poly-based coverings, with the ends open, and when a fine, nylon-mesh was used to cover the ends (*p* < 0.05). The poly-based covering also resulted in moderately higher ambient temperatures, which may have influenced beetle movement, including a “repellency effect” that encouraged beetles to exit the high tunnel structures. Although *P. japonica* adults are known to feed on raspberry flower clusters, including fruit, the majority (73–92%) of beetle feeding in this study was observed on the foliage. The impact of high tunnels on *P. japonica* are discussed within the context of developing sustainable Integrated Pest Management (IPM) programs for raspberry production.

## 1. Introduction

Raspberry continues to be one of the most popular fruit crops in the Midwestern U.S., where the demand for local produce is often greater than the supply. In Minnesota alone, the value of fresh-market raspberries was recently estimated at ~$1.5 million annually [1]. The Japanese beetle, *Popillia japonica* Newman (Coleoptera: Scarabaeidae), has been a damaging invasive species in the U.S. since 1916, attacking over 300 plant species (79 plant families), including high-value ornamental, residential turf, and fruit crops [2,3,4]. Among fruit crops, both floricane (summer bearing) and primocane (autumn bearing) raspberries are highly attractive to *P. japonica* adults. In Minnesota, the pest has been particularly damaging during the past decade; the beetles generate high levels of characteristic “skeletonizing” defoliation as well as some direct injury to fruit [2].

Raspberry production in the upper Midwest U.S. typically includes the use of alleyways (2–3 m width) or adjacent areas that are seeded into various grass species to manage weeds and minimize erosion [5]. The production system is highly advantageous to the univoltine life cycle of *P. japonica* in temperate regions [2]. Adult beetles are present from July to September, with oviposition occurring primarily in August-Sept. With ample raspberry foliage attracting adults for feeding and mating, the large grassy areas are highly preferred for oviposition and larval development [4,6]. In Minnesota, both established populations within local farms, and the dispersal of adult *P. japonica* into raspberry, serve as sources of infestation for fruit growers [2]. *Popillia japonica* is therefore typically present in raspberry from late June until October [7], resulting in about 12 weeks of feeding damage prior to, and during harvest. In turn, current year infestations may also serve to re-infest the grassy areas surrounding raspberries, leading to potential infestation with adults and larvae the following growing season. Each of these factors have led to increased insecticide use in summer and fall raspberry in Minnesota, where carbamate, pyrethroid and an organic-certified spinosyn are among those labeled for use in the U.S. [8,9].

Increasingly, the demand for establishing sustainable agriculture practices and the use of biologically based insecticides, particularly for organic production, has accelerated a need to examine alternatives to conventional broad-spectrum insecticides use [10]. The use of high tunnels has been growing in popularity over the last decade [11] and are typically used in the Upper Midwest for season extension or to improve quality of fruit through manipulation of microclimate, and reduced disease pressure [12,13]. The choice of coverings for high tunnel structures may include either a poly based plastic, most often for season extension, or fine- mesh netting, which can be used for pest exclusion. Recently the use of fine mesh netting as a pest management tactic has been documented for the ability to exclude pests, such as the invasive Spotted-wing Drosophila (*Drosophila suzukii*), from gaining access to fall raspberry [14,15] and wine grapes [16]. In northwest Italy, exclusion netting proved to be successful against a moth pest, *Grapholita molesta* (Busck), the brown marmorated stink bug, *Halyomorpha halys* (Stahl), and several additional pest species in nectarine orchards [17]. Additionally, in Pennsylvania, Cramer et al. [18] found that plastic coverings, which can modify ultraviolet light transmission, led to reduced populations of *P. japonica*, and subsequent feeding damage on raspberries; they examined several sources of plastic materials. We were therefore interested in assessing how some commonly used high tunnel coverings could repel or exclude *P. japonica* adults under Minnesota growing conditions and naturally occurring pest pressure. Here, we present data over a 3-yr period indicating that *P. japonica* are repelled, and numbers reduced, under plastic high tunnel coverings without ends installed and excluded once tunnels are enclosed.

## 2. Materials and Methods

**Figure 1:**
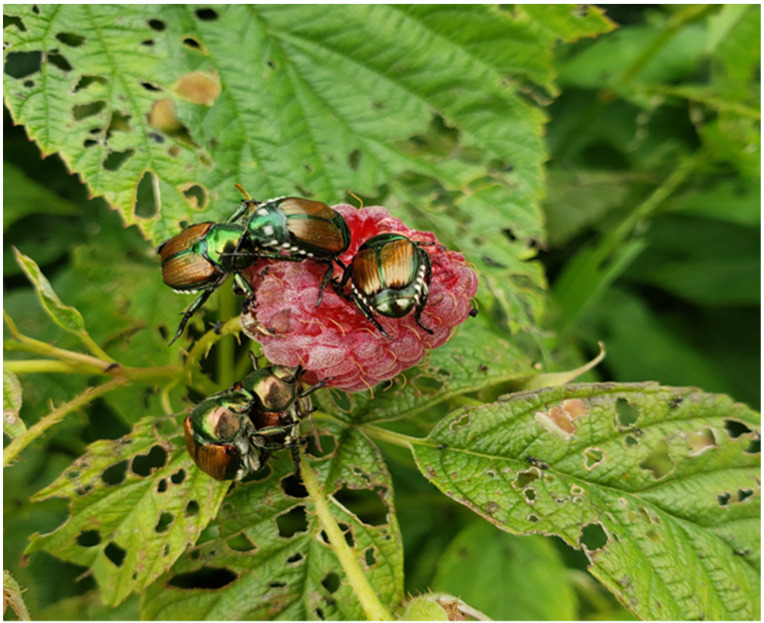
Adult *P. japonica* feeding injury on raspberry foliage and presence on primocane (autumn) raspberry fruit, Rosemount, MN, 2018 (A.G. Toninato, Univ. of Minnesota).

This study was conducted at the Rosemount Research and Outreach Center near Rosemount, MN, as part of a larger project to evaluate the presence of several insect species in the autumn-bearing red raspberry ‘Heritage’, grown under high tunnels. Preliminary observations in 2016 indicated that *P. japonica* numbers were typically suppressed under standard poly plastic covers, compared with adjacent open plots with no structure. We were therefore motivated to assess the potential population suppression in 2017–2019. Both foliar and flower samples (Figure 1) were taken for selected high tunnel treatments, compared to open plots, during each summer growing season (July Sept.).

**Figure 2:**
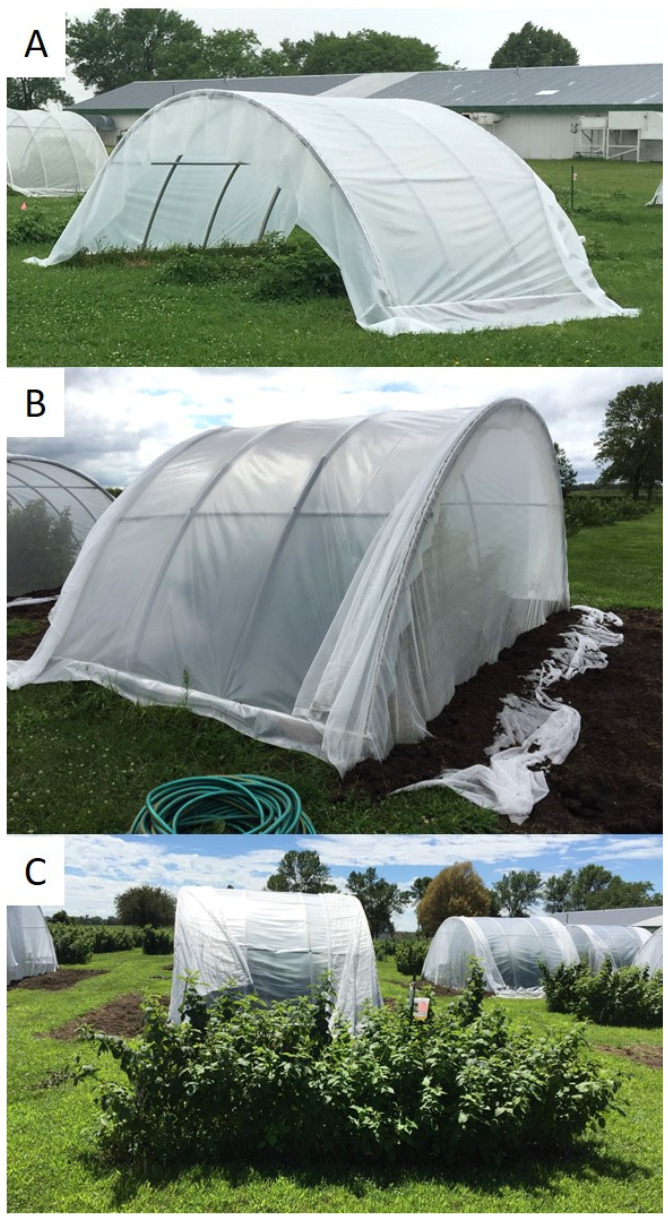
Primocane bearing red raspberry, ‘Heritage’, under high tunnels covered with standard poly plastic prior to installing mesh netting end pieces (**A**), after mesh netting ends were installed and buried with soil (**B**), and nearby open plots (untreated check) with no high tunnel structure or coverings (**C**); all treatments were arranged within a randomized complete block design, with 4 replications each, Rosemount Research & Outreach Center, MN, 2017–2019.

Experimental treatments consisted of (a) high tunnels covered with a standard GT-6 mil UV-stabilized poly (Poly-tex Inc., Castle Rock, MN, USA), (b) tunnels covered with Kool-Lite^®^ Plus (RKW Hyplast NV, Hoogstraten, Belgium), and (c) no high tunnel cover, referred to as the open plot (Figure 2). The three treatments were arranged in a randomized complete block design (RCBD), with 4 replications each. Coverings were placed on the structures initially to acclimate plants; after 1–2 weeks, fine-mesh netting, which consisted of 80-gram insect netting (Stone Wall Hill Farm LLC, Stephentown, NY, USA), ends were installed to enclose each tunnel [14]. Tunnel structures consisted of PVC ribs and purlins attached to a 5.1 cm × 20.3 cm × 304.8 cm wooden base-board on each side of the tunnel. Coverings were attached to the PVC structures using Wiggle Wire^®^ base and wire (Poly-Tex Inc., Castle Rock, MN, USA). Raspberry plantings were established in 2013, as described previously [14]. Within each high tunnel, bare root raspberries were planted in two rows that were 3.0 m long with 0.6 m spacing between plants and 1.5 m spacing between rows. High tunnels were 3.0 m long, 5.2 m wide and 2.1 m tall. For each year of the study, the treatments were arranged in a randomized complete block design (RCBD). In 2017–2019, weekly samples consisted of arbitrarily selecting 25 leaves and 25 flower clusters from across both rows within each plot. Sampling began 1–2 weeks prior to installing the ends of the tunnels in 2017 and 2018, respectively, and 5 weeks before ends were installed in 2019. To monitor temperature changes throughout the sampling period, Hobo U23 Pro V2 data recorders (Onset Comp. Corp., Bourne, MA, USA) were placed on a metal stake in three replicates of each treatment at 0.6 m above the ground and within the raspberry canopy. Beetle counts and temperature data were analyzed using a one-way analysis of variance (ANOVA) and a Tukey’s honest significant difference (HSD) test for mean separation, via the Statistical Analysis System [19]. Sampling data, for beetle location on plants, were analyzed using Chi-square with the expected hypothesis of equal (50:50) distribution of beetles between leaves and flower clusters [19].

## 3. Results and Discussion

In 2017 and 2018, prior to installing the ends on the high tunnels, the population of *P. japonica* was significantly higher in open plots compared to either the standard poly or Kool Lite poly treatments (Table 1 and Table 2). These findings were similar to those reported by Cramer et al. [18] and likely reflect the effect of plastic coverings on UV light transmission.

Observationally, the majority of beetles that were encountered under either poly treatment, were most often found on the very edge plants within a plastic covered high tunnel, suggesting they were being repelled by the conditions under the tunnel. On selected sample dates in 2019, open plot data recorded prior to closing the tunnels were not significantly higher than covered treatments; however, the open plots always had numerically higher counts (Table 3). Populations of *P. japonica* were somewhat lower in 2019 and may explain the lack of statistical differences for selected sample dates in July.

For all three years, however, once high tunnel ends were installed, *P. japonica* counts were consistently higher (*p* < 0.05) in open plots compared with either high tunnel treatment, and significantly higher in most cases (Table 1, Table 2 and Table 3). In summary, across all three years, beetle exclusion was evident for each of the high tunnel designs, where adult numbers were significantly lower than the adjacent open plots for 5 of 6, 4 of 5, and 5 of 8 sample dates (*p* < 0.05), in 2017–2019, respectively. In addition, where high tunnel poly treatments occurred without fine-mesh ends installed, a repulsion effect on beetle numbers was evident, compared to open plots, for the following sample dates evaluated: 8/1/2017, 7/16/2018, 7/30/2018, 7/10/2019, 8/7/2019, and 8/21/2019 (Table 1, Table 2 and Table 3). These results, with consistency across three years, provide confirmation that the use of plastic coverings and fine-mesh netting provides an adequate barrier to exclude or repel *P. japonica* adults, and is also known to be compatible for other insect pests of raspberry, such as *D. suzukii* (14,15).

One consideration for the use of high tunnels for pest exclusion, particularly for *P. japonica*, is that if tunnels are enclosed after insect pest activity begins and populations have infested the crop, the insects will be trapped inside the tunnel. Moreover, if infestations of *P. japonica* are present prior to the use or installation of high tunnels, *P. japonica* adults may emerge within tunnels from the previous years’ larval infestation in sod adjacent to the raspberries. In the present study, however, neither scenario was a concern. When observing where beetles were found feeding in the raspberry canopy, a clear preference was observed, where beetle occurrence and feeding injury was most frequent on foliage compared to flower clusters (Table 4).

Over the three years of sampling, a significantly higher number of beetles were found on leaves versus flower clusters (flower and fruit feeding combined), with percentages ranging from 73–92% on leaves, compared to 8–27% on flower clusters (Table 4; *p* < 0.0001). As *P. japonica* typically aggregate when feeding and mating [2,4], it is not unexpected that foliage could harbor more concentrated populations. Kowles and Switzer [20] found that female beetles were the pioneers of aggregations, and subsequently followed by males, supporting the concept that a feeding aggregation occurs which then leads to additional aggregations based on mating behavior. As raspberries go through a vegetative growth stage early in the growing season, which lasts until late August in Minnesota [21], the crop is an attractive host for summer infestations of *P. japonica* adults; the beetles therefore become well established on foliage prior to flower or fruit development. This information suggests the primary pest risk in Heritage raspberry is vegetative feeding injury, versus direct feeding to fruit. These results should be useful in developing sampling plans for management of *P. japonica* beetle populations in raspberry.

Temperatures under plastic covers were generally 0.5 to 1.5 °C warmer compared to open plots, with the average daily temperature significantly warmer (*p* < 0.05) in both the standard poly and Kool Lite treatments in both 2017 and 2018 (Table 5).

**Figure 3:**
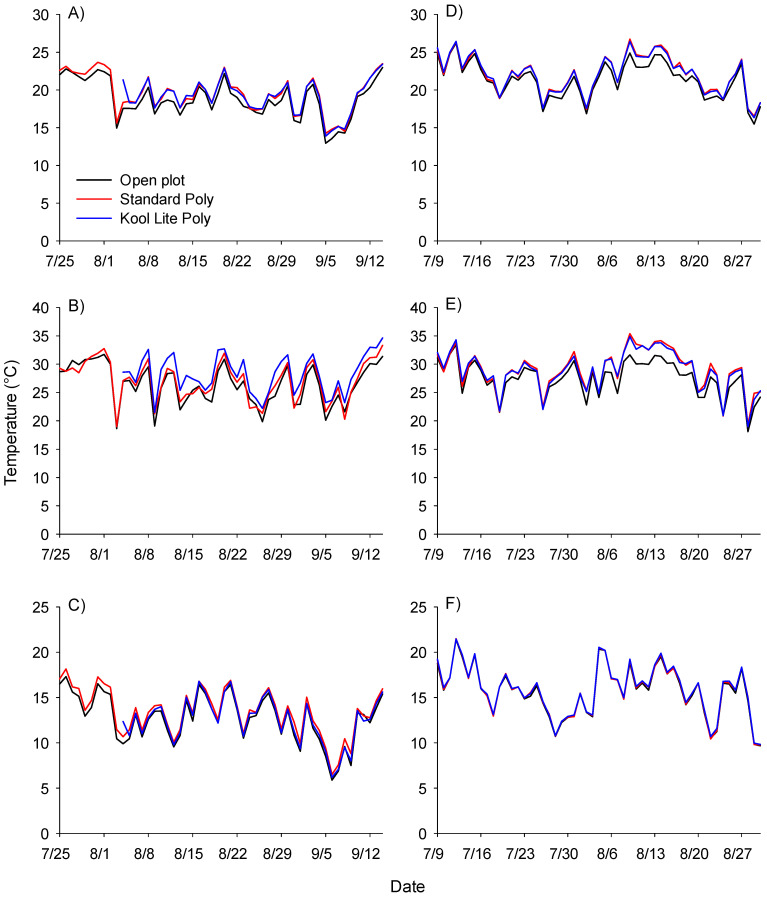
Temperature summary for various high tunnel treatments. Open plots are indicated with a black line, standard poly with a red line and Kool Lite poly with a blue line. Three replicates of temperature data were recorded with Hobo temperature data recorders and are summarized as mean average daily temperature in 2017 (**A**) and 2018 (**D**), mean maximum daily temperature in 2017 (**B**) and 2018 (**E**), and mean minimum daily temperature in 2017 (**C**) and 2018 (**F**).

While maximum and minimum daily temperatures were generally not significantly warmer in covered treatments compared with open plots, the trend was for warmer temperatures in covered treatments (Figure 3).

With a flight threshold of 25–27 °C [4] the plastic covered tunnels were likely to provide conditions for beetles to be more active, possibly moving in and out of the tunnels more often, leading to lower populations at any given time. However, Cramer et al. [18] determined there was a relationship between UV light transmission and beetle population density, where lower levels of UVA and UVB light transmission for plastic covers, was associated with lower beetle populations.

Despite the potential for several mechanisms involved in repelling *P. japonica* from poly-based high tunnels [18], it is apparent that plastic covers have the ability to reduce populations of adult beetles on foliage and subsequent defoliation. Along with this repellency, the exclusion of beetles, when using fine mesh netting ends in tandem with plastic covers, provides a high level of beetle reduction inside enclosed high tunnels, in some cases a 100% reduction. The trade-off in terms of the expense to implement high tunnel systems on a given farm, will depend on farm size (or specifically, berry production area), labor costs, and the decision to use Poly alone, or Poly with fine-mesh netting [1,14]. However, because the poly and mesh netting can be used for several years, both the initial and annual costs should be considered. Once more specific IPM guidelines are available, including validated, practical sampling plans [22] and economic thresholds for *P. japonica* on raspberry, we can begin to evaluate if these production practices are sufficiently effective to use alone or may still need to be combined with supplemental chemical controls [8,23]. Given the recent forecasts for continued range expansion of *P. japonica* in the U.S. and Europe [24], particularly under global climate change scenarios, it is imperative that novel IPM solutions for *P. japonica* continue to be investigated for high value agricultural crops [25].

## 4. Conclusions

To our knowledge, this is the first multi-year assessment of poly- and nylon-mesh based high tunnel designs to assess *P. japonica* exclusion for primocane raspberries. Our results are similar to a previous study that evaluated a variety of high tunnel designs for *P. japonica* [18]. The efficacy results in the present study, including the use of poly alone over the top of high tunnels, suggests there can be a substantial benefit to growers in managing the beetle, and also in reducing insecticide use. Ultimately, the use of high tunnel systems, as implemented by growers, will need to be evaluated at the farm level to ensure the sustainability of this approach.

## Figures and Tables

**Table 1:** Mean adult *P. japonica* counts in high tunnels covered with plastic and netting used for pest exclusion, ‘Heritage’ raspberry, Rosemount, MN, 2017.

Treatment	Mean (±SEM) Number Adult Beetles (Total for 25 Leaves and 25 Flower Clusters Per Plot)					
	Aug. 1 *	Aug. 10	Aug. 17	Aug. 24	Aug. 31	Sept. 7
Standard poly + ends; bumblebees added	5.50 ± 0.87 b	0.00 ± 0.00	1.50 ± 0.50 b	0.25 ± 0.25 b	0.00 ± 0.00 b	0.50 ± 0.29 b
Kool Lite poly + ends	3.50 ± 0.65 b	0.00 ± 0.00	0.00 ± 0.00 b	0.00 ± 0.00 b	0.00 ± 0.00 b	0.00 ± 0.00 b
Open Plot	43.50 ± 2.60 a	5.50 ± 3.20	14.50 ± 4.25 a	3.00 ± 1.00 a	9.25 ± 1.25 a	5.50 ± 2.02 a
		NS				

Means within columns followed by the same letter are not significantly different (*p* > 0.05), Tukey’s honest significant difference (HSD) test. Mean beetle counts were square root transformed for analysis; untransformed means are presented. NS = not significant, *p* > 0.05. * Mesh netting ends were installed on 8/8/17; therefore, the 8/1/17 sample was taken prior to tunnels being enclosed and all subsequent samples were after the tunnels had been enclosed.

**Table 2:** Mean adult *P. japonica* counts in high tunnels covered with plastic and netting used for pest exclusion, ‘Heritage’ raspberry, Rosemount, MN, 2018.

Treatment	Mean (±SEM) Number Adult Beetles (Total for 25 Leaves and 25 Flower Clusters Per Plot)				
	July 16	July 30 *	Aug. 9	Aug. 16	Aug. 23
Standard poly + ends; bumblebees added	3.25 ± 1.31 b	0.25 ± 0.25 b	0.50 ± 0.50 b	0.25 ± 0.25	0.25 ± 0.25 b
Kool Lite poly + ends	--	2.25 ± 1.03 b	0.00 ± 0.00 b	0.00 ± 0.00	0.00 ± 0.00 b
Open Plot	22.00 ± 2.58 a	12.75 ± 2.78 a	30.00 ± 8.51 a	1.00 ± 0.41	4.00 ± 1.47 a
				NS	

Means within columns followed by the same letter are not significantly different (*p* > 0.05), Tukey’s honest significant difference (HSD) test. Mean beetle counts were square root transformed for analysis; untransformed means are presented. NS = not significant, *p* > 0.05. * Mesh netting ends were installed on 8/2/18; therefore, the 7/16 and 7/30/18 samples were taken prior to tunnels being enclosed and all subsequent samples were after the tunnels had been enclosed.

**Table 3:** Mean adult *P. japonica* counts in high tunnels covered with plastic and netting used for pest exclusion, ‘Heritage’ raspberry, Rosemount, MN, 2019.

Treatment	Mean (±SEM) Number Adult Beetles (Total for 25 Leaves and 25 Flower Clusters Per Plot)							
	July 10	July 17	July 24	July 31	Aug. 7 *	Aug. 14	Aug. 21	Aug. 28
Standard poly + ends	--	--	--	--	--	1.00 ± 0.41 b	0.50 ± 0.50 b	0.50 ± 0.50 b
Standard poly—no ends	0.25 ± 0.25 b	3.50 ± 0.96	0.50 ± 0.29	0.50 ± 0.50	2.75 ± 0.48 b	3.25 ± 2.02 ab	2.25 ± 1.03 b	1.25 ± 0.48 ab
Open Plot	8.50 ± 2.96 a	13.75 ± 3.57	3.50 ± 1.19	6.50 ± 2.87	12.50 ± 1.32 a	13.75 ± 5.50 a	17.25 ± 5.88 a	6.75 ± 3.25 a
		NS	NS	NS				

Means within columns followed by the same letter are not significantly different (*p* > 0.05), Tukey’s honest significant difference (HSD) test. Mean beetle counts were square root transformed for analysis; untransformed means are presented. NS = not significant, *p* > 0.05. * Mesh netting ends were installed on 8/9/19; therefore, the 7/10, 7/17, 7/24, 7/31, and 8/7/19 samples were taken prior to tunnels being enclosed and all subsequent samples were after the tunnels had been enclosed.

**Table 4:** Distribution of adult *P. japonica* beetles during sampling of leaves and flower clusters of ‘Heritage’ autumn-bearing raspberries, Rosemount, MN, 2017–2019.

Year	Total Number of Adult Beetles					
	Per 25 Leaves	Per 25 Flower Clusters	Proportion on Leaves	Proportion on Flower Clusters	Χ^2^	*p* Value
2017 (n = 72)	270	100	0.73	0.27	77.2	*p* < 0.0001
2018 (n = 48)	189	16	0.92	0.08	144.3	*p* < 0.0001
2019 (n = 44)	218	29	0.88	0.12	143.1	*p* < 0.0001

Date ranges for data collection were as follows, 2017: Aug. 1 to Sep. 7; 2018: Jul. 30 to Aug. 23; 2019: Aug. 7 to Aug. 28. Chi-square conducted with expected hypothesis of equal (50:50) distribution of beetles on leaves vs. flower clusters (combined flowers + berry fruit).

**Table 5:** Mean temperatures one week before and after sample data collection for *P. japonica* adults in ‘Heritage’ autumn-bearing raspberries grown under high tunnel structures, Rosemount, MN, 2017 and 2018.

Year/Treatment	Mean Daily Temperature (°C)		
	Average	Maximum	Minimum
2017			
Standard poly + ends	19.56 ± 0.09 a	26.92 ± 0.25	13.37 ± 0.10 a
Kool Lite poly + ends	19.14 ± 0.03 b	28.27 ± 0.23	12.53 ± 0.03 b
Open Plot	18.84 ± 0.07 c	26.46 ± 0.73	12.74 ± 0.19 ab
		NS	
2018			
Standard poly + ends	22.09 ± 0.00 a	29.00 ± 0.15	15.81 ± 0.05
Kool Lite poly + ends	22.06 ± 0.15 a	28.87 ± 0.56	15.88 ± 0.03
Open Plot	21.36 ± 0.01 b	27.52 ± 0.06	15.70 ± 0.05
		NS	NS

Means within columns followed by the same letter are not significantly different (*p* > 0.05), Tukey’s honest significant difference (HSD) test. NS = not significant, *p* > 0.05.

## Data Availability

Raw data generated in this study are available upon request.
